# Risk factors of endometrial cancer in patients with endometrial hyperplasia: implication for clinical treatments

**DOI:** 10.1186/s12905-021-01452-9

**Published:** 2021-08-25

**Authors:** Jie Zhao, Yongting Hu, Yanan Zhao, Dongmei Chen, Tingfeng Fang, Miao Ding

**Affiliations:** 1 Department of Obstetrics and Gynecology, Fuping County Hospital, Weinan, China; 2 Department of Obstetrics and Gynecology, Sun Yat-sen Memorial Hospital of Sun Yat-sen University, No. 107 Road Yanjiang West, Guangzhou, China

**Keywords:** Endometrial cancer, Endometrial hyperplasia, Gynecology, Treatment, Care, Nursing

## Abstract

**Background:**

Endometrial hyperplasia (EH) is commonly-seen in the patients with endometrial cancer (EC), we aimed to evaluated the risk factors of EC in patients with EH, to provide evidence to the clinical prevention and treatment of EC.

**Methods:**

This study was a retrospective study design. EH patients confirmed by pathological examinations and treated with hysterectomy in our hospital from June 1, 2018 to February 28, 2021 were included. The clinical characteristics of EC and no-EC patients were compared and analyzed. Logistics regression analyses were conducted to evaluate the risk factors of EC in patients with EH.

**Results:**

A total of 228 EH patients were included, the incidence of EC in the EH patients was 31.58%. There were significant differences in the age, BMI, diabetes, hypertension and pathology of EH between EC and no EC groups (all *P* < 0.05), no significant differences in the hyperlipidemia, preoperative CA_125_, number of deliveries, menopause and endometrial thickness between EC and no EC groups were found (all *P* > 0.05). Logistic regression analyses indicated that age > 50 y (OR 3.064, 95% CI 1.945–5.931), BMI ≥ 25 kg/m^2^ (OR 2.705, 95% CI 1.121–3.889), diabetes (OR 3.049, 95% CI 1.781–5.114), hypertension (OR 2.725, 95% CI 1.108–3.431) and severe hyperplasia (OR 3.181, 95% CI 1.496–4.228) were the risk factors of EC in patients with EH (all *P* < 0.05).

**Conclusions:**

The risk of EC in EH patients is high, especially for those patients with age > 50 y, BMI ≥ 25 kg/m^2^, diabetes, hypertension and severe hyperplasia, special attentions should be paid for occurrence of EC and early diagnosis and early treatment are needed for those patients.

## Background

Endometrial cancer (EC) is one of the common malignant tumors in gynecology, and its incidence is increasing year by year, and is showing a trend of younger onset age [1]. It is generally believed that the continuous stimulation of estrogen without progesterone antagonism leads to endometrial hyperplasia and then cancerous transformation [2, 3]. Endometrial hyperplasia (EH) is a common gynecological endocrine disease, which mainly manifests as irregular vaginal bleeding, infertility, and even malignant transformation [4]. Endometrial dysplasia has certain tendency to become cancerous, and it is recognized as a precancerous lesion of EC with incidence of 23.15–29.08% [5, 6]. Therefore, early detection and intervention of EH is of great significance to improve the prognosis of patients.

Previous studies [7, 8] have shown that it’s very common that EH and EC can coexist in patients. The risk of endometrial dysplasia combined with endometrial cancer is as high as 25.76 to 59.22% [9, 10]. Clinically, there is a certain missed diagnosis rate in the diagnosis of EC [11]. How to identify high-risk patients with EC from patients with EH, is important to guide clinical treatment decisions and improve the prognosis of patients. Therefore, this present study analyzed the clinicopathological data of patients diagnosed with EH before hysterectomy, aimed to explore the high-risk factors of EC in patients with EH, to provide evidence support to the early prevention and clinical treatment of EC.

## Methods

### Ethics

This study was a retrospective study design. In this study, all methods were conducted in comply with the relevant guidelines and regulations. This present study had been verified and approved by the ethical committee of our hospital (approval number: MD10180068-2c), and written informed consents had been obtained from all the included patients.

### Patients

We selected EH patients treated with hysterectomy in our hospital from June 1, 2018 to February 28, 2021 as the study populations. The criterial for patient inclusion were: the endometrial thickness was determined by the B-ultrasound (endometrial thickness ≥ 4 mm) and pathological examinations, the ultrasound examinations of all selected patients were performed by the same group of sonographers in our hospital; patients underwent hysterectomy treatment in our hospital, and there were complete postoperative pathological reports; there was no history of hormone replacement therapy in the past 1 year. All consecutive patients meeting the selection criteria were included. Patients who did not agree to participant in this study and did not sign the inform consents were excluded.

### Data collection

We collected personal and treatment information of patients, including age, body mass index (BMI), diabetes, hypertension, hyperlipidemia, preoperative CA125 (U/ml) level, number of deliveries, menopause status, preoperative endometrial thickness, and the final pathological results.

### Treatment and follow-up

The diagnosis of EH referred to relevant standards and criteria [12, 13], and EH was divided into mild, moderate, and severe group according to the degree of disease. All patients underwent total hysterectomy in our hospital. According to the final pathological results after total hysterectomy, it was judged whether EC existed at the same time, and the diagnosis of EC referred to relevant standards. If the postoperative pathological examination results suggested that there was EC, further corresponding treatments were conducted. We conducted follow-up till the discharge of patients.

### Statistical method

We used SPSS 24.0 software to analyze the data, and the measurement data were expressed as mean ± standard deviation, and the comparison between the two groups used independent sample t test. Categorical variables were expressed as percentages, and Chi-square or Fisher exact probability tests were used for comparison between groups. We used logistic multiple regression analysis to explore high-risk factors related to EC. In this study, the difference was statistically significant with *P* < 0.05.

## Results

### Patients

A total of 228 EH patients were included, of whom 72 patients had EC, the incidence of EC in the EH patients was 31.58%. As presented in Table 1, there were significant differences in the age, BMI, diabetes, hypertension and pathology of EH between EC and no EC groups (all *P* < 0.05), no significant differences in the hyperlipidemia, preoperative CA_125_, number of deliveries, menopause and endometrial thickness between EC and no EC groups were found (all *P* > 0.05).

**Table 1:** The characteristics of included patients

Variables	EC group (n = 72)	No EC group (n = 156)	χ^2^/t	*P*
Age (y)	54.33 ± 9.26	46.12 ± 9.39	8.124	0.021
BMI (kg/m^2^)	27.16 ± 4.54	23.11 ± 5.95	6.101	0.036
Diabetes	39 (54.17%)	44 (28.21%)	1.114	0.019
Hypertension	52 (72.22%)	68 (43.59%)	1.271	0.038
Hyperlipidemia	17 (23.61%)	30 (19.23%)	1.855	0.076
Preoperative CA_125_ (U/ml)	21.69 ± 5.14	19.88 ± 4.24	1.232	0.095
Number of deliveries				
0	8 (11.11%)	20 (12.82%)	1.140	0.011
1	22 (30.56%)	48 (30.77%)		
2	37 (51.39%)	77 (49.36%)		
≥ 3	5 (6.94%)	11 (49.36%)		
Menopause	31 (43.06%)	63 (40.38%)	3.114	0.065
Pathology of EH				
Mild hyperplasia	8 (11.11%)	59 (37.82%)	1.892	0.008
Moderate hyperplasia	20 (27.78%)	85 (54.49%)		
Severe hyperplasia	44 (61.11%)	12 (7.69%)		
Endometrial thickness (mm)	10.27 ± 3.21	9.98 ± 2.02	1.774	0.102

*EC* endometrial cancer, *EH* endometrial hyperplasia

### Risk factors of EC in patients with EH

The variable assignments of multivariate logistic regression were presented in Table 2. As indicated in Table 3, logistic regression analyses indicated that age > 50 y (OR 3.064, 95% CI 1.945–5.931), BMI ≥ 25 kg/m^2^ (OR 2.705, 95% CI 1.121–3.889), diabetes (OR 3.049, 95% CI 1.781–5.114), hypertension (OR 2.725, 95% CI 1.108–3.431) and severe hyperplasia (OR 3.181, 95% CI 1.496–4.228) were the risk factors of EC in patients with EH (all *P* < 0.05).

**Table 2:** The variable assignment of multivariate logistic regression

Factors	Variables	Assignment
EC	Y	Yes = 1, no = 2
Age (y)	X_1_	≥ 50 = 1, < 50 = 2
BMI (kg/m^2^)	X_2_	≥ 25 = 1, < 25 = 2
Diabetes	X_3_	Yes = 1, No = 2
Hypertension	X_4_	Yes = 1, No = 2
Pathology of EH	X_5_	Severe hyperplasia = 1, moderate hyperplasia = 2, mild hyperplasia = 3

*EC* endometrial cancer, *BMI* body mass index, *EH* endometrial hyperplasia

**Table 3:** The logistic regression analysis on the risk factors of endometrial cancer in patients with endometrial hyperplasia

Variables	β	S${\overline{{\text{x}}}}$	OR	95% CI	*P*
Age > 50 y	0.121	0.287	3.064	1.945–5.931	0.012
BMI ≥ 25 kg/m^2^	0.115	0.206	2.705	1.121–3.889	0.027
Diabetes	0.106	0.152	3.049	1.781–5.114	0.009
Hypertension	0.142	0.187	2.725	1.108–3.431	0.034
Severe hyperplasia	0.119	0.105	3.181	1.496–4.228	0.019

*BMI* body mass index, *OR* odds ratio, *CI* confidence interval

## Discussions

The results of this present study have found that the incidence of EC in the EH patients was 31.58%, which is similar to previous reports [14, 15]. Besides, we have found that for patients with age > 50 y, BMI ≥ 25 kg/m^2^, diabetes, hypertension and severe hyperplasia, they may have higher risks of EC in patients with EH, early targeted preventions and interventions are needed for patients with those factors. The increase in estrogen levels in the body caused by various reasons continuously stimulates the proliferation of endometrial tissue [16]. The proliferated endometrial tissue is stimulated by a single estrogen for a long time without progesterone antagonism, which is likely to cause excessive endometrial cell proliferation [17]. Tumor cells infiltrate local blood vessels or necrosis and disintegration of their own tissues can cause bleeding [18]. The most common pathological type of endometrial cancer is endometrioid adenocarcinoma, which accounts for about 74.25 to 80.11% [19, 20]. Therefore, the early identification and treatment of EC in the EH patients is vital to the prognosis of EH patients.

The main cause is the long-term estrogen exposure of the endometrium and the lack of progesterone antagonism [21]. It is generally believed that endometrial cancer is more likely to occur in perimenopausal women [22]. Studies [23–25] have pointed out that age is a high-risk factor for endometrial dysplasia coexisting with EC. Previous study [26] has reported that endometrial hyperplasia patients aged 40–59 have an increased risk of endometrial cancer (OR 3.07, 95% CI 1.18–7.97); The risk is higher when the age is ≥ 60 (DR 6.65, 95% CI 1.75–25.3). The potential reasons may be that due to the perimenopausal age at which EC is likely to occur, which is related to the decline of the body's immune function [27]. It’s been reported that the incidence of EC under the age of 50 is 102 per 100,000, while the incidence of EC over the age of 50 has risen to 1374 per 100,000 [28]. The older the age, the more severe the disease and the lower the survival rate [29]. The results of this study also suggest that age ≥ 50 years old is a high-risk factor for the development of EC. Therefore, for the elderly, especially those with EH aged ≥ 48 years, they need to be treated with caution in clinical practice.

The pathological change of EH must be considered in the diseases progress. Among endometrial diseases, EH is the most common benign disease, and EC is one of the most common female reproductive system tumors, accounting for 20 to 30% of female reproductive tract malignant tumors [30]. In the past 20 years, the incidence of endometrial cancer has shown an obvious upward trend [31]. It is generally believed that endometrial lesions are a process in which the lesions continue to progress, that is, from not accompanied by EH to accompanied by EH, and finally progressing to well-differentiated EC [32, 33]. However, some scholars [21, 34] have reported that the incidence of EC in patients with endometrial hyperplasia is 12.18–46.34%. The results of this study also suggest that severe EH is also a high-risk factor for EC. However, the clinical reason for the failure to find coexisting EC before surgery may be that the preoperative endometrial sampling is mainly by diagnosing curettage, AEH lesions may be single or scattered lesions, which are affected by the experience of the surgeon, and there is a possibility of missed curettage [35, 36]. In addition, the diagnosis of well-differentiated EC is mainly based on the presence or absence of endometrial stroma, but it is sometimes difficult to determine [37]. This study has found that the risk of patients with endometrial cancer increase significantly with the severity of EH. Therefore, for patients whose endometrial biopsy pathology is severe EH, due to the high probability of EC, early alerts of EC are needed for those patients.

Several high-risk factors for EC should be concerned and get the attentions of clinical health workers. Studies [38, 39] have pointed out that obesity and diabetes are high-risk factors for EC. For every 5 kg/m^2^ increase in BMI, the risk of EC increases 1.6 times. Several studies [40, 41] have also shown that obesity in patients with endometrial cancer also increases the risk of endometrial cancer-related deaths. The reason is that adipose tissue is the source of estrogen in the body. Obesity causes excessive endogenous estrogen to act on the endometrium, leading to EH and EC. Studies [3, 42, 43] have shown that when the insulin resistance index is greater than or equal to 2.8809, the relative risks of endometrial hyperplasia and endometrial cancer are 35.22 and 30.59, respectively. It’s been found that when BMI ≥ 25 kg/m^2^, progesterone treatment is less effective and prone to recurrence. Therefore, weight control should be strengthened during treatment and follow-up. Simple hypertension does not increase the occurrence of endometrial cancer, but hypertension patients are often complicated by obesity and diabetes. Therefore, early control of weight, blood sugar and blood pressure are vital to improve the prognosis of patients.

It must be noted that the pathological types of EC with coexisting EH are hormone-dependent EC, more than 90% are well-differentiated cancers, about 40% of the lesions are confined to the endometrium, and about 50% are accompanied by superficial muscle layer infiltration, about 10% is accompanied by deep muscle layer infiltration [44]. Besides, there may be some immunohistochemical factors in predicting EC in EH patients. AT-rich interaction domain 1A (ARID1A) loss in premalignant EH is an accurate and almost perfectly specific prognostic marker for coexistent cancer [45]. Besides, it’s been found that PTEN loss in EH is a risk factor for EC, but is not reliable in predicting the risk of EC [46]. In this present study, four cases underwent lymph node resection after surgery, and the pathological examination results were negative. There was no recurrence or death due to cancer in the follow-up. Previous study [47] have found that after resection of the uterus due to EH are highly differentiated stage I cases, with only 6 cases of moderately differentiated cancer and 2 cases of poorly differentiated cancer.

## Conclusions

In conclusion, we have found that the incidence of EC in the EH patients was 31.58%, age > 50 y, BMI ≥ 25 kg/m^2^, diabetes, hypertension and severe hyperplasia are the independent risk factors of EC in patients with EH. For controllable high-risk factors, such as obesity, diabetes and hypertension, clinical education and intervention should be strengthened with more focus on changes in patients’ lifestyles, and early preventions are needed to reduce the development of EC.

## Data Availability

All data generated or analyzed during this study are included in this published article.

## Abbreviations

EC: Endometrial cancer
EH: Endometrial hyperplasia
BMI: Body mass index
OR: Odds ratio
CI: Confidence interval
